# Reevaluating C-Reactive Protein for Perioperative Risk Stratification: The Overlooked Role of Sleep Apnea in Cardiac Surgery Outcomes

**DOI:** 10.3390/biomedicines13102546

**Published:** 2025-10-18

**Authors:** Andrei Raul Manzur, Caius Glad Streian, Ana Lascu, Maria Alina Lupu, Horea Bogdan Feier, Stefan Mihaicuta

**Affiliations:** 1 Department of Doctoral Studies, “Victor Babes” University of Medicine and Pharmacy Timisoara, Eftimie Murgu Square No. 2, 300041 Timisoara, Romania; andrei.manzur@umft.ro; 2 Center for Research and Innovation in Precision Medicine of Respiratory Diseases, Department of Pulmonology, “Victor Babes” University of Medicine and Pharmacy Timisoara, Eftimie Murgu Square No. 2, 300041 Timisoara, Romania; stefan.mihaicuta@umft.ro; 3 Institute for Cardiovascular Diseases of Timisoara, Clinic of Cardiovascular Surgery, Gheorghe Adam Street No. 13A, 300310 Timisoara, Romania; horea.feier@umft.ro; 4 Department VI Cardiology, Cardiovascular Surgery Clinic, “Victor Babes” University of Medicine and Pharmacy Timisoara, Eftimie Murgu Square No. 2, 300041 Timisoara, Romania; 5 Department III Functional Sciences, Pathophysiology, “Victor Babes” University of Medicine and Pharmacy Timisoara, Eftimie Murgu Square No. 2, 300041 Timisoara, Romania; 6 Centre for Translational Research and Systems Medicine, “Victor Babes” University of Medicine and Pharmacy Timisoara, Eftimie Murgu Square No. 2, 300041 Timisoara, Romania; 7 Patogen Preventia, 300124 Timisoara, Romania; lupu.alina@umft.ro; 8 Clinical Laboratory, Institute of Cardiovascular Diseases, 300310 Timisoara, Romania; 9 Discipline of Parasitology, Department of Infectious Diseases, “Victor Babes” University of Medicine and Pharmacy, 300041 Timisoara, Romania; 10 Pulmonology Department, Infectious Diseases and Pulmonology Hospital “Victor Babes” Timisoara, 300310 Timisoara, Romania

**Keywords:** obstructive sleep apnea, cardiac surgery, postoperative atrial fibrillation, intubation time, C-reactive protein, perioperative risk stratification

## Abstract

**Background/Objectives:** C-reactive protein (CRP) is widely used as a marker of perioperative inflammation, but its predictive value for cardiac surgical outcomes remains uncertain. Obstructive sleep apnea (OSA), a prevalent and underrecognized comorbidity, may independently contribute to postoperative complications through non-inflammatory mechanisms. This study aimed to reevaluate the prognostic role of CRP and determine the clinical impact of OSA severity on postoperative recovery, focusing on new-onset atrial fibrillation (AF), prolonged intubation time, and postoperative CPAP/AIRVO use as indicators of respiratory burden. **Methods:** In this prospective cohort of 142 elective cardiac surgery patients, preoperative polysomnography and serial CRP measurements were obtained. Multivariable regression, mediation analysis, and propensity score matching (PSM) were performed to evaluate associations between OSA severity, CRP, and perioperative outcomes (AF, intubation time, CPAP/AIRVO use). **Results:** OSA severity independently predicted prolonged intubation (β = 1.74, *p* = 0.0019) and new-onset AF (β = 0.85, *p* = 0.004), even after excluding patients with preexisting arrhythmia. CRP showed poor discriminatory power as a standalone biomarker (AUC for IOT > 14 h = 0.445) and did not mediate OSA–outcome associations. However, CRP > 2.1 mg/dL doubled the odds of moderate-to-severe OSA (OR = 2.05, *p* = 0.041). A composite score integrating AHI, BMI, and postoperative CRP strongly correlated with postoperative respiratory support (*p* < 0.0001). **Conclusions:** OSA exerts a stronger and more consistent influence on perioperative outcomes than CRP, challenging reliance on CRP for risk stratification. Incorporating objective OSA screening and spirometry into preoperative assessment may enhance perioperative risk prediction and guide personalized management strategies.

## 1. Introduction

Obstructive sleep apnea (OSA) is a highly prevalent condition characterized by recurrent episodes of upper airway collapse during sleep, leading to intermittent hypoxemia, sleep fragmentation, and heightened sympathetic activity [1,2,3]. It affects 20–30% of adults in the general population and is particularly common among patients with cardiovascular comorbidities [4,5,6]. The incidence of OSA is elevated in individuals with coronary artery disease, heart failure, and arrhythmias, especially atrial fibrillation (AF) [7,8]. In the context of cardiac surgery, where structural and functional cardiac alterations are already present, the physiological disturbances of OSA may further amplify the risk of adverse perioperative outcomes [9,10]. Yet OSA remains substantially underdiagnosed in surgical populations, where preoperative evaluation often overlooks sleep-disordered breathing [9,11]. At the same time, perioperative risk stratification frequently relies on inflammatory biomarkers such as C-reactive protein (CRP), while the mechanistic contribution of OSA remains underexplored.

Cardiac surgery represents a setting where the physiological stresses of anesthesia, mechanical ventilation, and surgical trauma converge [12,13,14]. In untreated or unrecognized OSA, these factors may synergize to increase perioperative risk [15,16]. Postoperative atrial fibrillation (POAF), prolonged invasive and noninvasive ventilation, and heightened systemic inflammation are among the most frequent complications [17,18,19,20,21,22]. Mechanistically, intermittent hypoxia can induce myocardial remodeling, sympathetic activation, and oxidative stress, all known contributors to arrhythmogenesis and hemodynamic instability [23,24,25,26,27]. Repetitive desaturation may also drive systemic inflammation, impair wound healing, and compromise recovery in the postoperative period [28,29,30].

Previous studies have reported associations between OSA and unfavorable surgical outcomes, but many were retrospective or relied on indirect screening tools such as questionnaires [31,32,33,34]. Objective diagnostic methods, such as cardiorespiratory polygraphy, are needed to accurately stratify OSA severity in surgical patients [35]. Furthermore, the interplay between OSA and perioperative inflammatory biomarkers remains insufficiently characterized. Although CRP is widely used as a marker of systemic inflammation and perioperative risk [36,37,38,39,40], its predictive utility in patients with concomitant OSA undergoing cardiac surgery is uncertain.

Statins and other lipid-lowering therapies possess anti-inflammatory and endothelial-stabilizing effects beyond lipid reduction [41]. These pleiotropic properties may attenuate OSA-related systemic inflammation, particularly in the setting of cardiopulmonary bypass and ischemia–reperfusion injury [42,43,44]. While the perioperative impact of such therapies remains to be clarified, understanding how they interact with OSA-induced inflammation could inform management strategies [45,46,47].

In this prospective observational study, we integrated preoperative cardiorespiratory polygraphy into the perioperative evaluation of elective cardiac surgery patients. Our objectives were threefold: (1) to assess the incidence and severity of OSA; (2) to examine associations between OSA severity and major postoperative complications, including POAF, intubation time, and respiratory support requirements; and (3) to evaluate the prognostic role of CRP and explore whether lipid-lowering therapy modulates inflammatory responses across OSA severity levels. This comprehensive approach seeks to clarify the clinical and mechanistic relevance of OSA in the perioperative setting and to refine strategies for individualized risk stratification.

## 2. Materials and Methods

This study was conducted and reported in accordance with the STROBE (Strengthening the Reporting of Observational Studies in Epidemiology) guidelines for observational research [48]. A completed STROBE checklist is included with the manuscript submission.

### 2.1. Study Design and Ethical Approval

This prospective observational study was conducted over a three-year period (2022–2025) at the Institute of Cardiovascular Diseases Timișoara (Institutul de Boli Cardiovasculare Timișoara, IBCVT), a tertiary cardiac surgical center in western Romania, in clinical partnership with the Department of Pulmonology at the “Victor Babeș” Clinical Hospital for Infectious Diseases and Pulmonology, Timișoara. The study aimed to assess the incidence and clinical impact of obstructive sleep apnea in patients undergoing elective cardiac surgery, with a specific focus on postoperative complications, ventilatory outcomes, and inflammatory biomarker dynamics, all of which are shown in Supplementary Materials (Tables S1–S4). The study was approved by the Scientific Research Ethics Committee of the Victor Babeș University of Medicine and Pharmacy of Timișoara (UMFVBT), under approval number 101/19.12.2022, revised in 2025. The protocol complies with the principles of the Declaration of Helsinki, and written informed consent was obtained from all participants [49].

### 2.2. Patient Selection and Eligibility Criteria

An initial cohort of 180 patients scheduled for elective cardiac surgery was screened. Of these, 7 patients withdrew consent, 9 were postponed and initiated therapy for OSA (violating inclusion criteria), 19 were excluded due to major intraoperative or postoperative cardiovascular complications affecting ventilation time, and 3 declined the surgical intervention. The final study cohort consisted of 142 patients undergoing conventional cardiac surgery (CABG, AVR, MVR, or complex procedures) (see Figure S1 in the Supplementary Materials for the detailed patient flow diagram).

### 2.3. Preoperative Spirometry, Sleep Study and OSA Classification

All patients underwent a standardized preoperative evaluation within 24–48 h before surgery, including spirometry performed with the SpiroLab New system (MIR—Medical International Research, Rome, Italy) using a reusable turbine and integrated oximetry sensor, in accordance with ATS/ERS standards [50]. Overnight type III cardiorespiratory polygraphy was acquired with the Poligraf^®^ VitalNight PG system (Löwenstein Medical, Bad Ems, Germany), recording airflow, thoracoabdominal effort, pulse oximetry (SpO_2_), pulse rate, and body position. The apnea–hypopnea index (AHI) was computed automatically and verified by a trained scorer following AASM criteria [51]. OSA severity was classified by AHI as mild (5–<15 events/hour), moderate (15–<30 events/hour), and severe (≥30 events/hour).

Preoperative microbiological screening ensured the absence of active infection, verified through negative urine cultures, nasal/pharyngeal/inguinal swabs, and eradication of identified dental foci.

Clinical data collected included age, gender, BMI, smoking history, diabetes, hypertension, and left ventricular ejection fraction (EF). Medication history included statin and non-statin lipid-lowering agents. Biochemical parameters, including C-reactive protein and lipid profile (total cholesterol, HDL, LDL, and triglycerides), were assessed. Preoperative CRP as well as the lipid profile were measured on the morning of surgery, while postoperative CRP was quantified at 24 h following the procedure.

### 2.4. Laboratory Testing and Biochemical Assays

C-reactive protein was quantified using the Dimension clinical chemistry system with the CRP Extended Range (RCRP) Flex reagent cartridge (Siemens Healthcare Diagnostics, Tarrytown, NY, USA). This particle-enhanced turbidimetric immunoassay provides an analytical range of 0.05–250.0 mg/L, with a functional sensitivity of 0.15 mg/L and a precision coefficient of variation (CV) < 10% at concentrations as low as 0.5 mg/L. Although high-sensitivity CRP (hsCRP) assays, such as the Siemens CardioPhase hsCRP method, are designed for accurate detection of low-grade systemic inflammation and offer a lower limit of detection (0.1 mg/L) with a validated clinical range of 0.1–15.0 mg/L, both methods are technically appropriate within the overlapping range of 0.3–15.0 mg/L. Although high-sensitivity CRP (hsCRP) assays are optimized for detecting subtle elevations in systemic inflammation, particularly in cardiovascular risk stratification, the present study prioritizes capturing acute-phase inflammatory responses in the perioperative setting. Standard CRP assays, such as the Siemens RCRP used here, are specifically validated for higher concentration ranges (up to 250 mg/L) and maintain acceptable precision (CV < 10%) even at low-to-moderate values (≥0.5 mg/L).

CRP was modeled both as a predictor (e.g., of POAF and intubation time) and as an outcome (e.g., in relation to spirometry and statin use).

Given that postoperative inflammation often results in CRP levels far exceeding 15 mg/L, the upper limit of most hsCRP assays, the use of a standard CRP method ensures greater dynamic range and reliable quantification of peak inflammatory responses. Importantly, the analytical overlap between CRP and hsCRP (e.g., 0.3–15 mg/L) allows standard CRP to remain informative even for patients with lower baseline levels.

Thus, the choice of standard CRP provided both the necessary range and resolution to assess preoperative variability and postoperative escalation without sacrificing analytic validity or interpretability.

As per CDC/AHA guidelines, CRP values < 1 mg/L were interpreted as low cardiovascular risk, 1–3 mg/L as moderate risk, and >3 mg/L as indicative of high cardiovascular risk.

Serum total cholesterol, HDL-cholesterol, LDL-cholesterol, and triglycerides were measured on the Dimension^®^ clinical chemistry system (Siemens Healthcare Diagnostics, Tarrytown, NY, USA) using Flex^®^ reagent cartridges (Siemens Healthcare Diagnostics Inc., Newark, DE, USA). Assay principles were enzymatic CHOD-PAP for total cholesterol, enzymatic GPO-PAP (after lipase hydrolysis) for triglycerides, direct homogeneous PEG-cholesterol esterase/oxidase for HDL-C, and a direct homogeneous selective-detergent method for LDL-C. Analytical measurement ranges were HDL-C 3–150 mg/dL (0.08–3.89 mmol/L), total cholesterol 50–600 mg/dL, LDL-C 5–300 mg/dL, and triglycerides 15–1000 mg/dL. Calibrations and internal quality control followed routine laboratory procedures.

### 2.5. Ventilatory Management and Clinical Outcomes

Mechanical ventilation parameters included total ventilation time and mode (IPPV, SIMV, CPAP). Post-extubation respiratory support included CPAP and high-flow nasal therapy (AIRVO™ 2, Fisher & Paykel Healthcare, Auckland, New Zealand), delivering heated, humidified oxygen via nasal cannula at adjustable flow rates. Postoperative atrial fibrillation (POAF) was also tracked and recorded as a clinical endpoint.

### 2.6. Sample Size and Statistical Power

A priori power analysis indicated that a minimum of 106 patients was required to detect a difference in postoperative atrial fibrillation incidence (45% vs. 20%) between severe and no/mild OSA groups (α = 0.05, 80% power, Cohen’s h = 0.54). The final cohort of 142 patients exceeded this threshold, ensuring sufficient power for core comparisons and multivariable modeling.

### 2.7. Clinical Endpoints

The pre-specified clinical endpoints were: (I) new-onset POAF and (II) postoperative intubation time, selected a priori as the two perioperative outcomes most consistently linked to OSA in prior literature. The need for postoperative CPAP/AIRVO support was analyzed as a secondary clinical endpoint. The sample-size calculation was based on detecting differences in POAF, given the larger expected effect size; analyses of IOT and CPAP/AIRVO were otherwise pre-specified and interpreted alongside multivariable, mediation, and propensity-score methods.

### 2.8. Bias Minimization and Blinding

Selection bias was minimized by recruiting patients consecutively from all eligible individuals undergoing elective cardiac surgery during the study period, based on predefined objective criteria. Measurement bias was addressed through standardized data collection protocols, with independent and blinded assessment of cardiorespiratory polygraphy results, biochemical markers, and postoperative outcomes.

All patients with known diabetes, hypertension, or a history of atrial fibrillation received treatment prior to presentation at our clinic, ensuring baseline management of major comorbidities. History of atrial fibrillation was specifically recorded to distinguish pre-existing cases from new-onset postoperative atrial fibrillation, which served as a key outcome.

Confounding was addressed using multivariate regression and propensity score matching, incorporating covariates such as age, BMI, diabetes, hypertension, and preoperative AF status. Missing data, primarily associated with clinical severity, were handled via multiple imputation using predictive mean matching. Uniform in- hospital follow-up was maintained across all patients to limit differential outcome detection.

### 2.9. Statistical Analysis

All statistical analyses were conducted using R version 4.3.0 and Python 3.10 [52,53], with relevant packages including dplyr, ggplot2, mice, MatchIt, scikit-learn, statsmodels, and Pingouin. Data normality was assessed using the Shapiro–Wilk test; non-normally distributed variables (e.g., CRP, intubation time) were analyzed using non-parametric tests (Mann–Whitney U, Kruskal–Wallis) or log-transformed where appropriate. Multivariable logistic and linear regression models were employed to evaluate associations between OSA severity and postoperative outcomes, adjusting for age, BMI, hypertension, diabetes, left ventricular ejection fraction, and statin use. Model assumptions, including linearity, homoscedasticity, and multicollinearity, were verified prior to interpretation, with all variance inflation factor (VIF) values below 5.

Subgroup analyses, interaction terms (e.g., AHI × spirometry), and mediation models (using non-parametric bootstrapping, n = 5000) were performed to explore underlying mechanisms. A composite risk index combining z-scored AHI, CRP, and BMI was constructed and tested for predictive value across respiratory and arrhythmic outcomes. Propensity score matching (1:1 nearest neighbor, caliper = 0.2 SD) was conducted separately for moderate vs. no/mild and severe vs. no/mild OSA groups, with covariate balance assessed via standardized mean differences and visualized using Love plots.

Rationale for bootstrapping: Several endpoints (e.g., intubation time, ΔCRP) and model residuals exhibited skew and small-to-moderate strata across OSA severity. We therefore used nonparametric bootstrapping (5000 resamples) to obtain bias-reduced standard errors and percentile confidence intervals for regression and mediation estimates, avoiding reliance on large-sample normal approximations and improving interval coverage under non-normality and heteroscedasticity. Resampling was performed at the individual level with replacement, preserving the observed covariate structure.

Additional technical details and statistical outputs are available in Supplementary Materials.

## 3. Results

### 3.1. Baseline Characteristics

All statistical procedures, including model diagnostics, threshold modeling, and composite index evaluation, were pre-specified in the publicly registered Statistical Analysis Plan, available at the Open Science Framework (https://doi.org/10.17605/OSF.IO/2GY34) (accessed on 21 July 2025). The study cohort consisted of 142 patients who underwent elective cardiac surgery and completed preoperative cardiorespiratory polygraphy for sleep apnea classification. Baseline clinical, biochemical, surgical, and ventilatory variables were analyzed across four strata of obstructive sleep apnea severity: no OSA, mild, moderate, and severe. This section outlines the demographic composition, comorbidity burden, surgical distribution, and relevant preoperative laboratory markers, serving as a foundation for interpretation of postoperative outcome analyses.

The study was powered to detect differences in postoperative atrial fibrillation between OSA severity groups; prolonged intubation time and postoperative CPAP/AIRVO use were pre-specified as complementary clinical outcomes, reflecting the two complications most consistently associated with OSA in prior literature.

#### 3.1.1. Full Cohort Overview

The study cohort (N = 142) comprised 102 males (71.8%) and 40 females (28.2%), with 54.9% reporting a smoking history and 62.0% demonstrating normal spirometry. Surgical procedures included CABG (44.4%), AVR (22.5%), MVR (16.9%), and complex operations (43.7%). Postoperative complications increased with OSA severity: atrial fibrillation rose from 19.4% in mild OSA to 60.0% in severe OSA, while CPAP/AIRVO use increased from 3.2% to 20.0%. Severe OSA patients also exhibited the highest mean preoperative CRP (5.7 ± 10.4 mg/dL) and intubation time (16.9 ± 7.0 h). Lipid profiles showed no consistent gradient, though higher cholesterol levels in the no-OSA group likely reflected lower statin use. These findings underscore the greater comorbidity burden and respiratory support needs in severe OSA, justifying stratified outcome analyses (Supplementary Materials).

#### 3.1.2. Descriptive Statistics with IQRs for Key Variables

**Figure 1:**
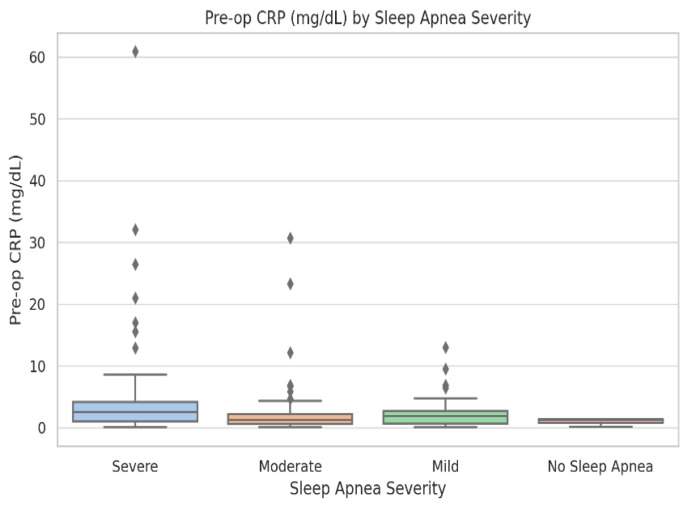
Boxplot of preoperative C-reactive protein (mg/dL) across sleep-apnea severity levels: No OSA (n = 38), Mild (n = 46), Moderate (n = 41), and Severe (n = 17). Boxes show medians and interquartile ranges; whiskers represent 1.5 × IQR, and dots denote outliers. Kruskal–Wallis test: *p* = 0.018. CRP values and variability increase progressively with apnea severity.

**Figure 2:**
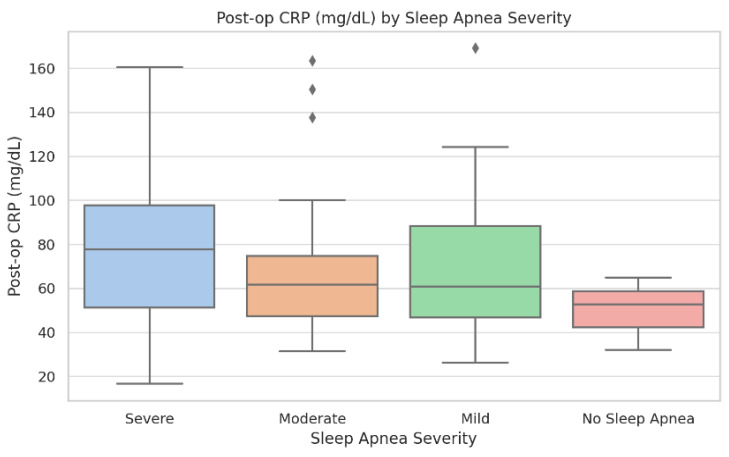
Postoperative C-reactive protein (mg/dL) across OSA severity categories. Boxplots display median, interquartile range, and outliers. Group sizes: No OSA (n = 38), Mild (n = 46), Moderate (n = 41), and Severe (n = 17). Kruskal–Wallis test: *p* = 0.012. Patients with severe OSA demonstrate higher CRP values and greater inter-individual variability.

**Figure 3:**
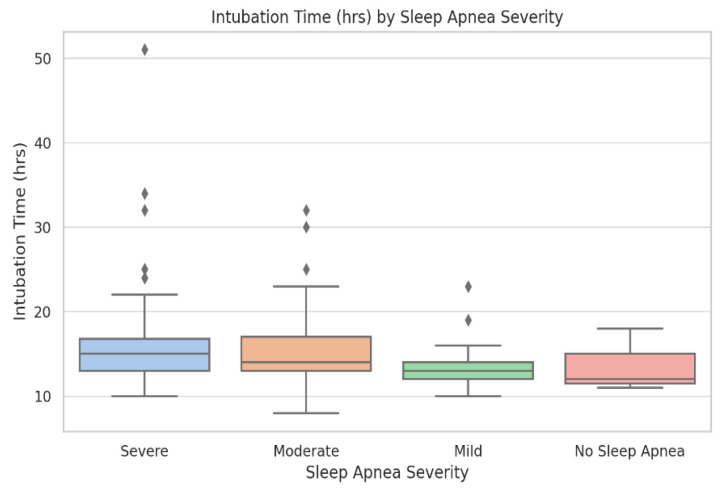
Postoperative intubation time (hours) across OSA severity categories. Boxplots display median, interquartile range, and outliers. Group sizes: No OSA (n = 38), Mild (n = 46), Moderate (n = 41), and Severe (n = 17). Kruskal–Wallis test: *p* = 0.006. Intubation duration increases progressively with apnea–hypopnea index (AHI) category and is more variable in severe OSA.

Descriptive statistics for demographic, inflammatory, and ventilatory variables across OSA severity groups are provided in Table 1 and in the Supplementary Materials Tables S1–S3. Boxplots of pre- and postoperative CRP (Figure 1 and Figure 2) and intubation time (Figure 3) demonstrate rising medians and greater variability with increasing OSA severity. Rates of new-onset atrial fibrillation and postoperative CPAP/AIRVO use followed a similar trajectory, reinforcing the progressive functional burden of severe OSA. These descriptive trends support the need for the stratified analyses presented below.

Baseline demographic, clinical, and laboratory characteristics stratified by OSA severity are summarized in Table 1. The groups were generally comparable in terms of age, sex, and comorbidities. Significant differences were observed for pre-operative CRP, postoperative atrial fibrillation, and intubation time, which increased progressively with OSA severity (*p* < 0.05). Complete descriptive and inferential statistics, including IQRs and raw values, are available in Supplementary Tables S2 and S3 and in the raw dataset (Table S1).

### 3.2. Core Associations Between Sleep Apnea Severity and Outcomes

#### 3.2.1. AHI and Postoperative Atrial Fibrillation

**Figure 4:**
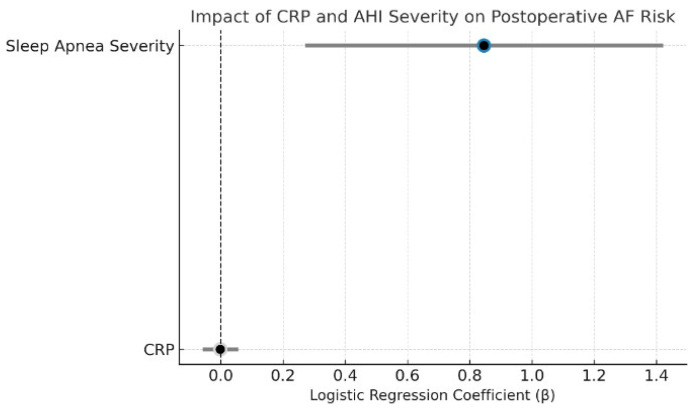
Dot plot of CRP and sleep apnea severity effects on postoperative atrial fibrillation. This plot displays the logistic regression coefficients (β) and 95% confidence intervals from a model predicting new-onset postoperative AF. Sleep apnea severity was a significant independent predictor (*p* = 0.004), while CRP showed no association (*p* = 0.94). Color coding reflects statistical significance: blue (AHI = significant), gray (CRP = non-significant). The dashed vertical line represents the null effect (β = 0).

In multivariable logistic regression excluding patients with preoperative AF, OSA severity was a significant independent predictor of new-onset postoperative AF (β = 0.85, *p* = 0.004), whereas CRP was not associated with risk (β = −0.0022, *p* = 0.94). Model discrimination was acceptable (AUC = 0.74; Figure 4). The complete regression output, including coefficients, confidence intervals, and *p*-values for all covariates, is presented in Supplementary Table S4.

**Figure 5:**
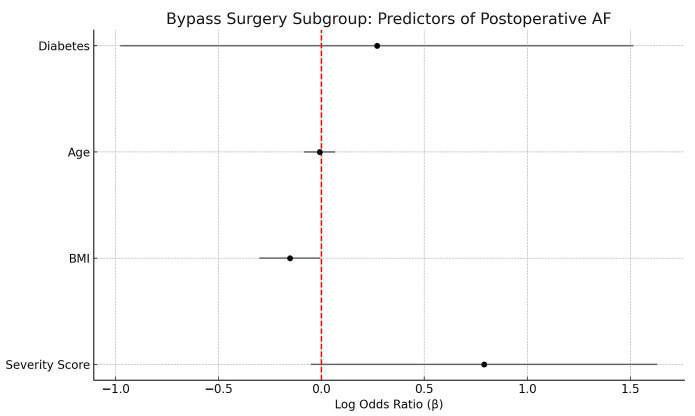
Forest plot showing regression coefficients (β) and 95% confidence intervals for predictors of postoperative atrial fibrillation in the Bypass Surgery subgroup. Sleep apnea severity (ordinal score) demonstrated a suggestive positive association with AF risk (*p* = 0.066), while BMI was inversely associated with AF (*p* = 0.042). Vertical red line indicates the null effect (β = 0).

Subgroup analyses confirmed that OSA severity predicted AF in aortic valve replacement (β = 1.45, *p* = 0.014) and complex procedures (β = 1.05, *p* = 0.024), with a borderline effect in CABG (β = 0.79, *p* = 0.066). In CABG, BMI was inversely associated with AF (β = −0.15, *p* = 0.042), while other covariates were non-significant (Figure 5).

Although the distribution of surgical procedures (CABG, AVR, MVR, and complex operations) varied slightly across OSA severity groups, inter-group differences were not statistically significant (χ^2^ *p* = 0.38). Furthermore, multivariable and propensity-matched analyses were adjusted for surgical type, confirming that the associations between OSA severity and both postoperative atrial fibrillation and intubation time remained robust after accounting for procedural category. Because valve procedures inherently carry a higher baseline AF risk, subgroup analyses were also performed (Figure 5), which confirmed that OSA severity remained an independent predictor within individual surgical subsets.

An exploratory interaction with bypass time (Supplementary Figure S2) suggested a synergistic effect in severe OSA but it was not statistically significant. Overall, OSA severity is a robust and independent predictor of postoperative AF, particularly in higher-risk surgical groups, whereas CRP showed no predictive value.

#### 3.2.2. AHI and Intubation Time

**Figure 6:**
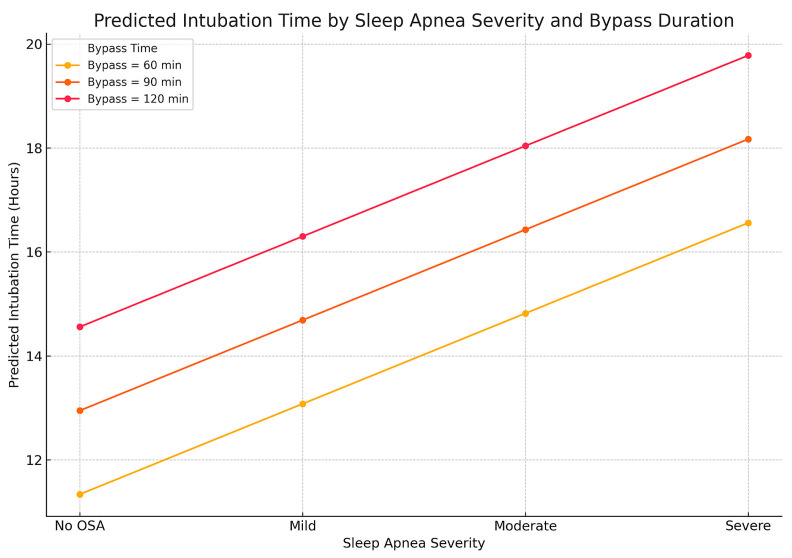
Relationship between sleep apnea severity, bypass time, and predicted duration of mechanical ventilation after cardiac surgery. The three lines represent different durations of cardiopulmonary bypass during surgery (60, 90, and 120 min). For each bypass time, intubation time increases step by step as OSA severity progresses from “No OSA” to “Severe.” The combined effect is most pronounced in patients with both a longer bypass time and severe OSA, emphasizing the value of sleep apnea screening in surgical planning.

Postoperative intubation time is an important indicator of respiratory recovery. We evaluated the association between OSA severity and IOT using both linear and logistic regression. In the linear model, each incremental increase in OSA severity was associated with a 1.74 h increase in IOT (β = 1.74, *p* = 0.0019). Bypass time was also significant (β = 0.054, *p* < 0.0001). Other variables, including age, BMI, and comorbidities, were not significant (Figure 6).

**Figure 7:**
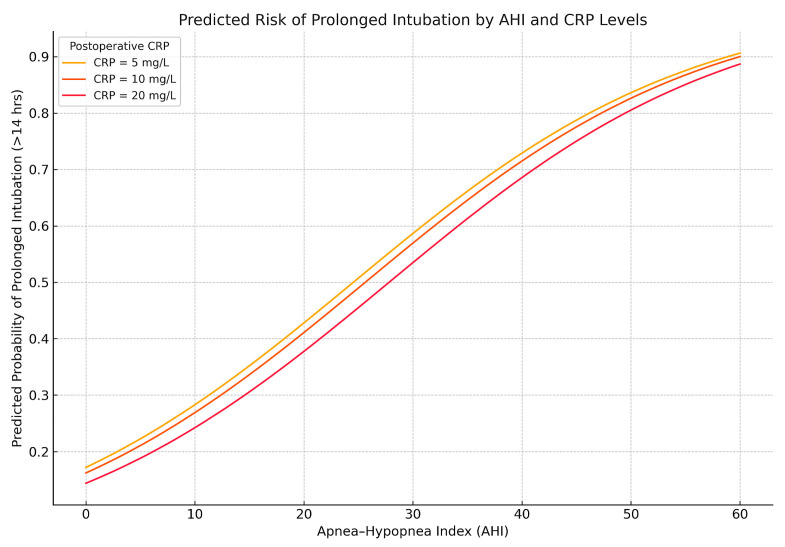
Predicted probability of prolonged intubation (>14 h) based on AHI and postoperative CRP. Predicted probabilities from a logistic regression model demonstrate increasing risk of prolonged mechanical ventilation with higher apnea–hypopnea index. Interestingly, lower CRP values are associated with slightly higher predicted risk, reflecting the inverse relationship observed in the model. This may reflect clinical decisions to extubate earlier in patients with elevated CRP due to perceived resilience or low reintubation risk.

In logistic regression (cutoff: IOT > 14 h), AHI remained a strong predictor (β = 0.064, *p* < 0.001), while postoperative CRP was inversely associated (β = −0.014, *p* = 0.044), possibly reflecting extubation decisions in patients with high inflammation but stable clinical status. These relationships are illustrated in Figure 7.

These findings reinforce that OSA severity is a consistent and independent predictor of prolonged intubation following cardiac surgery.

#### 3.2.3. AHI and CPAP/AIRVO Use

**Figure 8:**
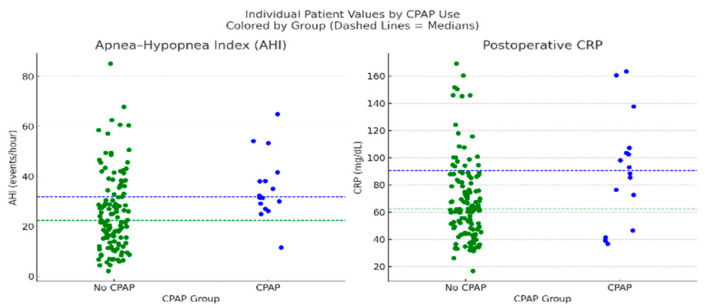
Dot plots comparing individual patient values for AHI and postoperative CRP between patients who received postoperative CPAP/AIRVO support (n = 16) and those who did not (n = 126). Each dot represents a single patient. Dashed lines indicate group medians, blue for the CPAP group and green for the non-CPAP group. Mann–Whitney U tests showed significant differences for both parameters: AHI (*p* = 0.0045) and CRP (*p* = 0.0173). Patients who received CPAP support exhibited higher median AHI (31.9 vs. 22.4) and CRP levels (90.65 vs. 62.35 mg/dL), supporting the role of respiratory burden and inflammation in guiding postoperative ventilatory support decisions.

We evaluated predictors of postoperative CPAP or AIRVO use, a proxy for respiratory burden. As shown in Figure 8, univariate comparisons showed higher AHI (median 31.9 vs. 22.4; *p* = 0.0045) and CRP (median 90.65 vs. 62.35 mg/dL; *p* = 0.0173) in the CPAP group.

**Figure 9:**
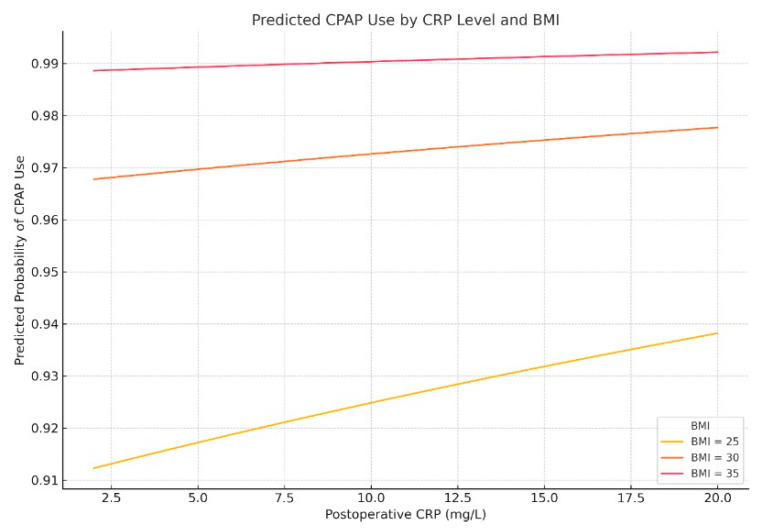
Predicted probability of postoperative CPAP use by postoperative CRP level and BMI. Multivariable logistic regression modeling shows that the likelihood of receiving postoperative CPAP or AIRVO support increases with both elevated CRP levels and higher BMI.

In the multivariable model presented in Figure 9, postoperative CRP (β = 0.021, *p* = 0.032) and BMI (β = 0.212, *p* = 0.006) were independent predictors of postoperative CPAP use. The corresponding odds ratios were 1.02 (95% CI, 1.00–1.04) for CRP and 1.24 (95% CI, 1.06–1.44) for BMI, indicating that higher inflammatory burden and greater adiposity independently increased the likelihood of postoperative ventilatory support. AHI was not significant (*p* = 0.31), suggesting that CRP and adiposity may mediate its effects.

These results suggest that postoperative CPAP decisions are more closely aligned with markers of inflammation and metabolic stress than with AHI alone. CRP and BMI may therefore serve as valuable adjuncts in respiratory management planning.

### 3.3. CRP as an Independent Predictor

**Figure 10:**
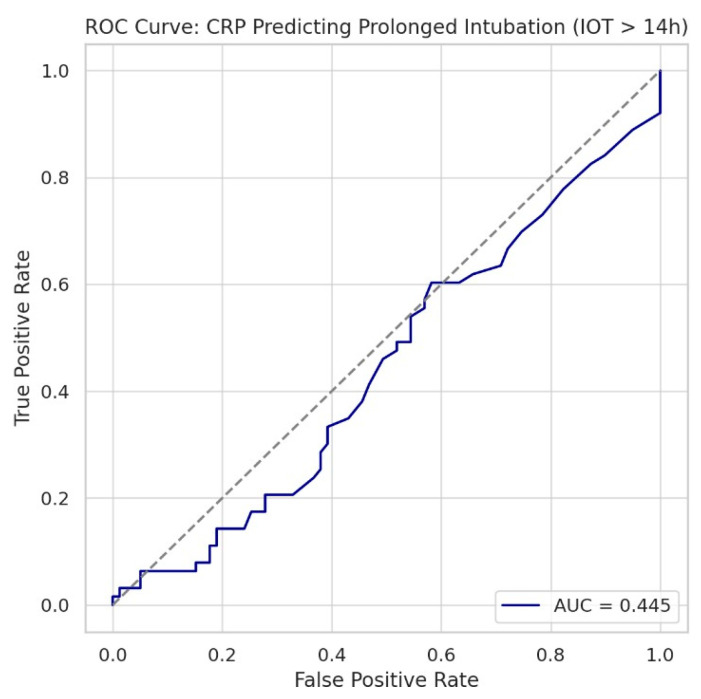
Receiver operating characteristic curve evaluating the ability of C-reactive protein to predict prolonged postoperative intubation time (>14 h). The area under the curve was 0.445, indicating that CRP performs worse than chance as a standalone predictor in this context. The diagonal dashed line represents a random classifier (AUC = 0.50). These results support the conclusion that CRP is not a reliable biomarker for predicting postoperative ventilatory duration.

We evaluated CRP as a predictive biomarker using ROC (Figure 10) and threshold analyses. Additional exploratory analyses, including the association of statin therapy with lower preoperative CRP, the influence of age and ventilatory phenotype on postoperative CRP, and the borderline relationship of ΔCRP with postoperative CPAP use (*p* = 0.056), are presented in Supplementary Figures S3–S8.

To further investigate the clinical utility of CRP in OSA detection, we conducted a systematic threshold sweep analysis evaluating CRP cutoffs (1.0–5.0 mg/dL) against increasing AHI severity levels (AHI > 15, >20, >25, >30). This approach aimed to identify whether discrete CRP thresholds are predictive of higher OSA burden.

**Figure 11:**
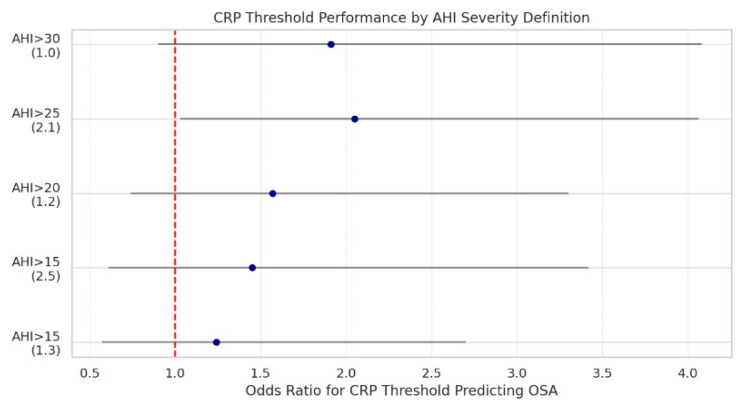
Odds ratios and 95% confidence intervals for predicting moderate-to-severe OSA at various apnea–hypopnea index thresholds, using selected CRP cutoff values. Each point represents the odds ratio (OR) for a binary CRP threshold at a corresponding AHI severity definition. For example, CRP > 2.1 mg/dL significantly predicted AHI > 25 with an OR of 2.05 (*p* = 0.041). The red dashed line denotes the null association (OR = 1). As OSA severity increases, CRP becomes a more meaningful predictor, suggesting that inflammation may play a larger role in advanced disease states.

However, CRP > 2.1 mg/dL was modestly associated with moderate-to-severe OSA (AHI > 25) (OR = 2.05, *p* = 0.041), suggesting potential clinical utility as a simple, widely available screening adjunct for sleep-disordered breathing in surgical patients (Figure 11).

Taken together, these findings indicate that although CRP reflects systemic inflammation, its predictive value for postoperative complications is limited. Statin therapy was associated with lower preoperative CRP, while postoperative CRP was more strongly influenced by age and ventilatory phenotype than by OSA severity. ΔCRP correlated with BMI and showed only a borderline relationship with postoperative CPAP use (*p* = 0.056).

Importantly, a threshold of CRP > 2.1 mg/dL was significantly associated with moderate-to-severe OSA (OR = 2.05, *p* = 0.041), suggesting a potential role as a simple screening adjunct. Overall, CRP should not be used in isolation for perioperative risk stratification but may contribute as a contextual biomarker when integrated with anthropometric and physiologic parameters.

### 3.4. Composite and Interaction Models

#### Combined Risk Score (AHI + BMI + CRP)

To capture the multidimensional physiologic burden associated with postoperative complications, we constructed a composite risk score by summing z-standardized values of the apnea–hypopnea index, postoperative CRP, and body mass index.

**Figure 12:**
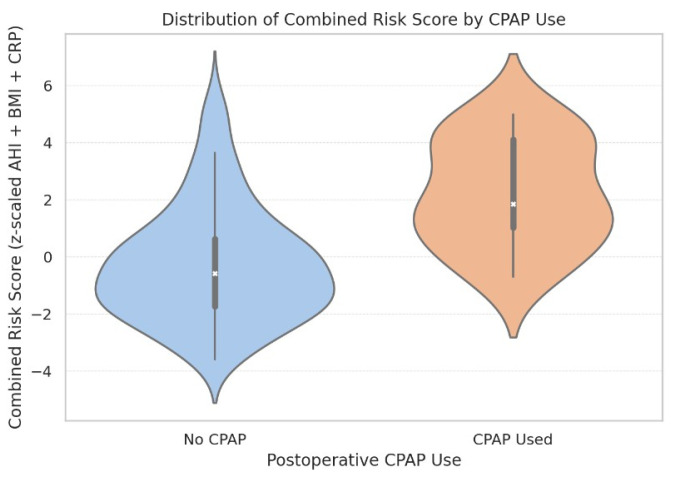
Violin plot showing the distribution of combined risk scores stratified by postoperative CPAP use. Internal boxes indicate median and interquartile ranges. The width of each violin reflects the score distribution density. CPAP recipients exhibited a markedly higher median score (1.85) compared to non-CPAP patients (−0.58), supporting the score’s utility in perioperative risk stratification.

As shown in Figure 12, this combined score was significantly associated with new-onset postoperative atrial fibrillation (ρ = 0.25, *p* = 0.0029) and effectively discriminated between patients who required postoperative CPAP support versus those who did not (median 1.85 vs. −0.58, *p* < 0.0001). However, the score was not predictive of intubation time as a continuous variable (*p* = 0.231).

These findings suggest that aggregated physiological and inflammatory burden, as captured by this composite index, may help identify patients at higher risk of postoperative respiratory support needs, though its predictive value for ventilation duration is limited.

To further evaluate whether systemic inflammation mediates the association between OSA severity and IOT, a mediation model was constructed with postoperative CRP as the mediator.

AHI was a significant predictor of IOT (β = 0.0982), confirming a direct relationship. However, the indirect effect via CRP was negligible (point estimate = 0.0000; 95% CI: −0.005 to 0.0044), with an estimated proportion mediated of 0%.

This analysis indicates that the effect of sleep apnea severity on postoperative intubation time is not mediated by systemic inflammation, and likely reflects mechanisms intrinsic to OSA such as pharyngeal collapsibility or ventilatory control instability.

Together, these analyses confirm that OSA severity exerts direct effects on postoperative outcomes, whereas CRP contributes little explanatory value beyond a minor role in integrated risk scores.

### 3.5. Propensity Score-Matching Analyses

To further isolate the impact of sleep apnea severity on postoperative outcomes, two separate propensity score-matching (PSM) analyses were conducted. These analyses excluded patients with known preoperative atrial fibrillation to ensure that all AF cases represented new-onset postoperative events. Patients with moderate or severe OSA were independently matched 1:1 to patients with no or mild OSA based on age, BMI, diabetes, hypertension, and smoking status. Post-matching covariate balance was verified using standardized mean differences (SMDs), with all covariates falling below the conventional 0.1 threshold.

#### 3.5.1. Moderate vs. No/Mild OSA

In the moderate OSA cohort (AHI 15–30), 49 matched pairs were analyzed after excluding patients with preoperative AF. Patients with moderate sleep apnea had significantly higher rates of postoperative atrial fibrillation (44.9% [22/49] vs. 20.4% [10/49]; *p* = 0.0085), as well as prolonged intubation times (median: 14.0 vs. 12.0 h; *p* = 0.0074). These differences remained statistically significant despite covariate adjustment.

**Figure 13:**
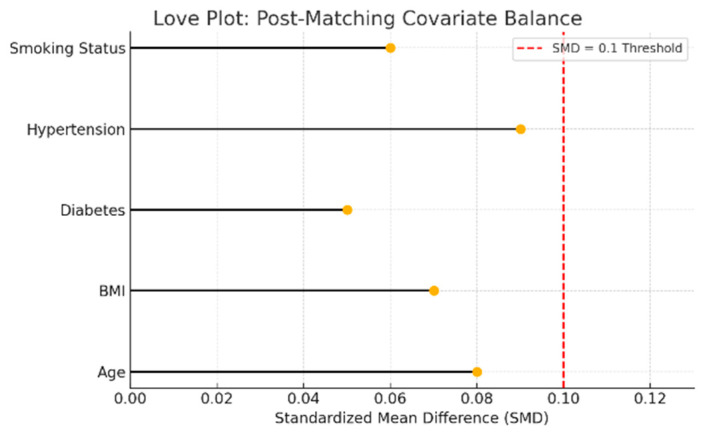
Love plot showing standardized mean differences (SMD) for matching covariates between moderate and no/mild sleep apnea groups. Each dot represents the post-matching SMD for a covariate. The red dashed line at 0.1 indicates the conventional threshold for acceptable covariate balance. Values below this threshold suggest that matching successfully reduced baseline differences.

Post-matching balance diagnostics are shown in Figure 13, which demonstrates that all covariates used in the matching procedure achieved standardized mean differences below the 0.1 threshold, indicating excellent match quality.

#### 3.5.2. Severe vs. No/Mild OSA

The severe OSA cohort (AHI > 30) included 41 matched patients versus 46 with no/mild OSA after excluding preoperative AF cases. Severe OSA was associated with a markedly increased risk of postoperative AF (58.5% [24/41] vs. 8.7% [4/46]; *p* < 0.001) and longer intubation times (median: 15.0 vs. 13.0 h; *p* < 0.001). These findings confirm that even after rigorous covariate matching, severe sleep-disordered breathing exerts a significant adverse effect on postoperative recovery.

**Figure 14:**
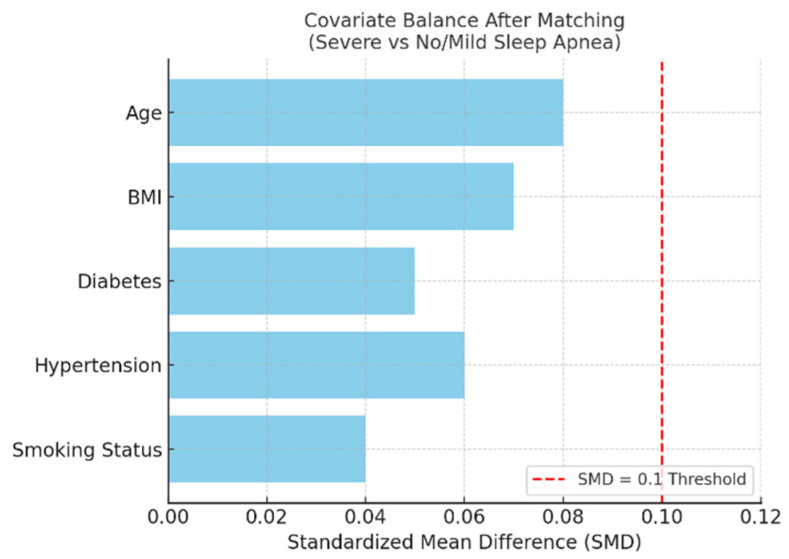
Bar plot showing post-matching standardized mean differences (SMDs) for covariates used in propensity score matching between patients with severe and no/mild sleep apnea, excluding those with preoperative atrial fibrillation. All covariates achieved acceptable balance, with SMD values below the conventional 0.1 threshold (red dashed line), indicating successful adjustment for baseline differences.

Figure 14 illustrates covariate balance between matched groups, confirming post-match equivalence across all variables included in the model.

Taken together, these propensity score-matched analyses confirm that both moderate and severe OSA independently increase the risk of postoperative AF and prolonged ventilation. These robust findings underscore OSA as a modifiable perioperative risk factor, supporting routine screening and targeted respiratory management in cardiac surgery pathways.

## 4. Discussion

### 4.1. Summary of Key Findings

This prospective observational study demonstrated that OSA is an independent predictor of prolonged intubation time and new-onset postoperative atrial fibrillation, two complications most consistently linked to OSA in the perioperative literature, while analyses of other outcomes were exploratory. Across multivariable regression, mediation analysis, and propensity score matching, OSA severity (apnea–hypopnea index) consistently predicted both prolonged intubation and new-onset postoperative atrial fibrillation, even after adjusting for cardiometabolic comorbidities, incorporating pulmonary and lipid parameters, and excluding patients with preoperative AF.

By contrast, CRP showed only a modest correlation with OSA but did not independently predict AF or intubation, nor did it mediate the effect of AHI. Preoperative statin therapy was associated with lower baseline CRP, confirming its anti-inflammatory potential, though without measurable impact on clinical endpoints. Spirometry revealed that patients with mixed or distal ventilatory impairments had greater risk of prolonged intubation, highlighting the additive role of baseline pulmonary dysfunction.

Overall, these findings emphasize the primacy of sleep-disordered breathing and ventilatory mechanics over systemic inflammation in shaping perioperative outcomes, and extend prior reports [9,54,55] by integrating objective polygraphy, spirometry, and biomarker profiling into a multidimensional risk stratification framework.

### 4.2. Comparison with Prior Literature

The link between OSA and postoperative atrial fibrillation observed here is consistent with prior reports on the arrhythmogenic effects of sleep-disordered breathing [56,57,58]. Intermittent hypoxemia and sleep fragmentation are known to drive atrial remodeling, sympathetic activation, and systemic inflammation, key mechanisms of AF pathogenesis [59,60,61]. Large retrospective and registry-based studies have also reported increased AF risk in OSA patients undergoing cardiac surgery [62,63,64], though most relied on clinical suspicion or questionnaires rather than formal polygraphy, limiting diagnostic precision.

Our study strengthens this evidence by using objective preoperative cardiorespiratory polygraphy within a prospective design and by excluding patients with pre-existing AF, thereby improving causal inference. The inclusion of spirometry, rarely assessed in surgical OSA cohorts, revealed independent contributions of ventilatory abnormalities to postoperative burden.

We also evaluated systemic inflammation and statin therapy. Although CRP offered limited predictive value, its association with OSA severity and suppression by statins support its role as a physiologic marker rather than a standalone risk stratifier. The anti-inflammatory and antioxidant properties of statins, while not directly linked to improved outcomes here, warrant further study in targeted interventions.

Taken together, these methodological advances enhance the mechanistic depth and translational relevance of our findings, while reinforcing the consensus that OSA is a significant, underrecognized determinant of perioperative morbidity in cardiac surgical patients.

### 4.3. Mechanistic Interpretations

The adverse impact of OSA on surgical outcomes likely reflects interacting cardiopulmonary and neurohumoral pathways [16,46,65]. Intermittent hypoxia induces oxidative stress, endothelial dysfunction, and myocardial remodeling, creating a pro-arrhythmic substrate [66,67,68]. Recurrent arousals with sympathetic surges further destabilize autonomic tone, predisposing to postoperative atrial fibrillation [69]. Fluctuating intrathoracic pressures and systemic inflammation impose additional atrial stress and impair pulmonary compliance, prolonging ventilatory support [70].

Our findings align with these mechanisms. OSA severity (AHI) consistently predicted both prolonged intubation and new-onset AF, independent of CRP, indicating that inflammation does not fully explain these effects. Spirometric abnormalities, particularly mixed and distal obstructive patterns, were strong predictors of prolonged ventilation, highlighting how compromised baseline pulmonary function amplifies OSA-related risk, an underexplored factor in prior studies.

Exploratory analyses also suggested a modulatory role of statin therapy and systemic inflammation. While statin use did not directly influence outcomes, it was associated with lower preoperative CRP, supporting its recognized anti-inflammatory and antioxidant actions.

Together, these results support a multifactorial model: OSA heightens perioperative vulnerability through hypoxia-driven autonomic dysregulation and ventilatory instability, with inflammation and metabolic regulation serving as secondary modifiers rather than primary drivers.

### 4.4. Clinical Implications

This study carries direct clinical implications for perioperative management in cardiac surgery. The consistent association between OSA severity and adverse outcomes, namely prolonged intubation and new-onset AF, supports integrating OSA screening into standard preoperative evaluation, especially for high-risk patients. Objective assessment with cardiorespiratory polygraphy is more reliable than questionnaires or clinical suspicion, which often miss sleep-disordered breathing in surgical populations.

Our findings also highlight the value of incorporating spirometry into perioperative risk profiling. Ventilatory abnormalities emerged as strong predictors of prolonged intubation, underscoring the combined effect of OSA and compromised pulmonary function. Mediation analyses confirmed that CRP did not explain OSA’s impact, reinforcing the primacy of ventilatory mechanics and autonomic dysregulation. Still, a CRP threshold > 2.1 mg/dL modestly identified moderate-to-severe OSA, suggesting it could serve as a pragmatic adjunct when access to sleep studies is limited.

Exploratory results further suggest a role for statins and other anti-inflammatory strategies in perioperative modulation, though targeted trials are needed before clinical adoption.

**Figure 15:**
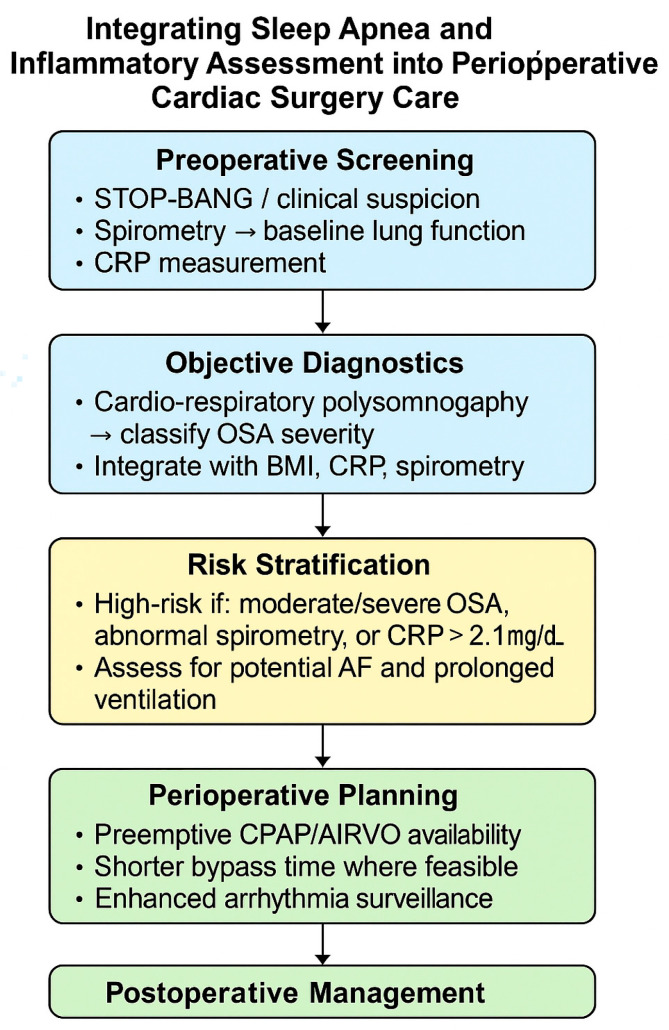
Integrating sleep apnea and inflammatory assessment into perioperative cardiac surgery care. This schematic illustrates a proposed clinical decision pathway for incorporating obstructive sleep apnea screening and inflammatory profiling into the perioperative management of cardiac surgery patients. The pathway begins with preoperative screening using STOP-BANG, spirometry, and CRP measurement, followed by objective diagnostics through cardio-respiratory polysomnography. Combined evaluation of OSA severity, spirometric pattern, and CRP levels enables risk stratification to identify patients at higher risk of arrhythmia and prolonged ventilation. These insights guide individualized perioperative planning, emphasizing proactive CPAP or AIRVO support, optimized bypass time, and enhanced postoperative monitoring. Arrows (→) indicate sequential diagnostic steps from assessment to classification.

Collectively, these findings argue for multidisciplinary perioperative pathways that integrate sleep medicine, pulmonary evaluation, and individualized ventilatory planning. Such approaches could enable earlier identification of vulnerable patients, guide tailored postoperative support (e.g., CPAP or AIRVO), and reduce complications from unrecognized OSA. Importantly, widely used surgical risk models (EuroSCORE II, STS) omit sleep-disordered breathing; incorporating validated OSA assessment may improve their predictive precision and enhance patient-specific care (Figure 15).

### 4.5. Strengths and Limitations

This study has several strengths. It employed a prospective design with preoperative cardiorespiratory polygraphy, ensuring precise classification of OSA severity, an improvement over prior studies based on suspicion or questionnaires. The integration of spirometry, perioperative inflammatory markers, and medication history enabled multidimensional profiling and novel insights into how respiratory function and systemic inflammation intersect in surgical outcomes. Methodologically, the use of multivariable regression, mediation analysis, and propensity score matching provided a triangulated analytic framework that reduced confounding and enhanced internal validity. Analyses were considered to be complete cases because the analysis variables contained no missing data, and we employed nonparametric bootstrapping (5000 resamples) to obtain robust confidence intervals under skewed outcomes.

The study’s limitations must also be acknowledged. The sample size, while adequate for primary analyses, limited power for subgroup and interaction tests. Single-night cardiorespiratory polygraphy may not fully capture night-to-night variability in OSA severity. CRP, though widely used, does not encompass the full spectrum of immunologic or oxidative stress pathways relevant to perioperative physiology. While statin use was recorded, adherence, dosing, and timing were not standardized, which may have diluted potential effects. While bootstrapping improves interval estimation under non-normality, it does not address bias from unmeasured confounding and may be sensitive to small-cell instability in subgroup or interaction analyses.

Finally, this was a single-center study in a specialized cardiac surgical unit, which may limit generalizability to other surgical populations or healthcare systems. Despite these limitations, the combination of prospective design, objective respiratory assessment, and rigorous statistical methodology provides a strong foundation for future multicenter validation and interventional research.

### 4.6. Future Directions

The findings of this study highlight several priorities for future research. First, randomized trials and prospective interventional studies are warranted to test whether perioperative optimization of sleep apnea, through CPAP therapy, supplemental oxygen, or pharmacologic modulation of autonomic tone, can reduce postoperative complications in cardiac surgery.

Second, our results support evaluating the integration of routine spirometry and cardiorespiratory polygraphy into preoperative protocols, particularly for patients with metabolic comorbidities or those undergoing high-risk procedures. Future work should assess not only diagnostic accuracy but also feasibility, cost-effectiveness, and long-term outcomes of such pathways.

Third, the biomarker landscape requires expansion beyond CRP. Serial perioperative measurements of inflammatory and oxidative stress markers, such as interleukin-6, myeloperoxidase, or circulating microRNAs, may provide superior discriminatory power compared with static CRP values and clarify the mechanistic links between OSA and perioperative vulnerability.

Fourth, pharmacologic strategies deserve further evaluation. Although statin therapy was associated with lower baseline CRP in this cohort, it did not independently affect clinical outcomes. Nevertheless, the known anti-inflammatory and antioxidant effects of statins, together with exploratory findings, support testing intensified statin regimens or adjunctive antioxidant therapies in stratified trials of patients with polygraphy-confirmed OSA.

Finally, external validation in larger, multicenter cohorts is needed to confirm generalizability and refine risk stratification models. Incorporating OSA severity, spirometric phenotypes, and perioperative biomarker trajectories into existing surgical scores may enhance predictive precision and enable personalized perioperative planning.

Collectively, these directions emphasize the potential of combining objective sleep apnea screening, pulmonary function testing, and biomarker profiling to advance individualized perioperative care and reduce morbidity in patients with undiagnosed or undertreated OSA.

## 5. Conclusions

This prospective observational study demonstrated that obstructive sleep apnea is an independent predictor of two key postoperative outcomes in cardiac surgery, prolonged intubation time and new-onset atrial fibrillation, which are the complications most consistently linked to OSA in prior literature. These associations remained significant across multivariable, mediation, and propensity score-matched analyses, underscoring the clinical relevance of sleep-disordered breathing in the perioperative setting.

C-reactive protein showed limited predictive capacity in this context. Although a threshold of >2.1 mg/dL modestly identified moderate-to-severe OSA, CRP neither mediated nor independently predicted postoperative complications. Statin therapy was associated with lower baseline CRP but did not translate into improved outcomes. Thus, CRP appears to serve as a contextual adjunct marker rather than a standalone predictor of risk.

Integrating objective OSA diagnostics and spirometry into preoperative evaluation may strengthen current cardiac-surgical risk models, which often overlook sleep-disordered breathing. Such integration could facilitate earlier identification of vulnerable patients, guide individualized perioperative planning, and inform decisions regarding respiratory support strategies.

In summary, this study, which is powered for atrial fibrillation and includes pre-specified analyses of intubation time and postoperative respiratory support, suggests that physiologic measures of sleep-disordered breathing and pulmonary function provide more clinically relevant insights than inflammatory markers alone. Recognition and management of OSA should become an integral component of cardiac-surgical care to improve perioperative outcomes.

## Figures and Tables

**Table 1:** Baseline demographic, surgical, and biochemical characteristics stratified by OSA severity. It includes demographic, surgical, comorbid, inflammatory, and lipid-related parameters. Categorical variables are shown as counts and percentages. Continuous variables are shown as mean ± standard deviation.

Variable	Total	No Sleep Apnea	Mild	Moderate	Severe
Smoking History	78 (54.9%)	0 (0.0%)	16 (51.6%)	34 (58.6%)	28 (56.0%)
Male Sex	102 (71.8%)	1 (33.3%)	23 (74.2%)	41 (70.7%)	37 (74.0%)
Female Sex	40 (28.2%)	2 (66.7%)	8 (25.8%)	17 (29.3%)	13 (26.0%)
Known AF (pre-op)	22 (15.5%)	0 (0.0%)	4 (12.9%)	9 (15.5%)	9 (18.0%)
Post-op AF	64 (45.1%)	1 (33.3%)	6 (19.4%)	27 (46.6%)	30 (60.0%)
Normal Spirometry	88 (62.0%)	3 (100.0%)	21 (67.7%)	35 (60.3%)	29 (58.0%)
Bypass Surgery	63 (44.4%)	1 (33.3%)	16 (51.6%)	23 (39.7%)	23 (46.0%)
AVR Surgery	32 (22.5%)	1 (33.3%)	2 (6.5%)	18 (31.0%)	11 (22.0%)
MVR Surgery	24 (16.9%)	0 (0.0%)	4 (12.9%)	10 (17.2%)	10 (20.0%)
Complex Procedures	62 (43.7%)	2 (66.7%)	12 (38.7%)	28 (48.3%)	20 (40.0%)
Diabetes	51 (35.9%)	1 (33.3%)	12 (38.7%)	18 (31.0%)	20 (40.0%)
Hypertension	129 (90.8%)	2 (66.7%)	28 (90.3%)	52 (89.7%)	47 (94.0%)
Post-extubation CPAP/AIRVO Use	15 (10.6%)	0 (0.0%)	1 (3.2%)	4 (6.9%)	10 (20.0%)
Statin Use	122 (85.9%)	3 (100.0%)	26 (83.9%)	51 (87.9%)	42 (84.0%)
Bypass Time (min)	107.0 ± 36.1	104.7 ± 8.5	95.8 ± 22.7	112.7 ± 39.2	107.5 ± 39.0
Intubation Time (hrs)	15.5 ± 5.4	13.7 ± 3.8	13.2 ± 2.6	15.7 ± 4.7	16.9 ± 7.0
Pre-op CRP (mg/dL)	3.7 ± 7.2	1.0 ± 0.7	2.6 ± 2.9	2.7 ± 5.1	5.7 ± 10.4
HDL Cholesterol (mg/dL)	41.5 ± 15.9	44.5 ± 7.7	41.8 ± 17.6	42.1 ± 16.2	40.5 ± 15.2
LDL Cholesterol (mg/dL)	75.8 ± 34.4	124.0 ± 41.9	74.1 ± 41.8	71.6 ± 29.7	78.8 ± 32.6
Total Cholesterol (mg/dL)	141.0 ± 46.2	190.3 ± 49.0	139.5 ± 54.3	135.6 ± 41.7	145.1 ± 44.9
Ejection Fraction (%)	47.3 ± 7.5	45.0 ± 13.2	48.7 ± 5.9	47.2 ± 8.2	46.5 ± 7.3

## Data Availability

The preregistered statistical analysis plan is publicly available in the Open Science Framework (OSF) repository at https://doi.org/10.17605/OSF.IO/2GY34 (accessed on 21 July 2025). The original contributions presented in this study are included in the article. Further inquiries can be directed to the corresponding authors.

## Supplementary Materials

The following supporting information can be downloaded at https://www.mdpi.com/article/10.3390/biomedicines13102546/s1. Table S1. Raw sleep apnea cardiac surgery dataset (de-identified). Table S2. Baseline characteristics stratified by OSA severity (AHI classification): demographic, surgical, ventilatory, inflammatory, and lipid variables. Table S3. Descriptive statistics by OSA severity: means, SDs, and IQRs for continuous variables. Table S4. Multivariable logistic regression model output for new-onset postoperative atrial fibrillation (full coefficients and 95% CIs). Figure S1. Participant flow diagram (screening, exclusion, final cohort n = 142). Figure S2. Interaction between OSA severity and bypass time for postoperative atrial fibrillation risk. Figure S3. CRP distribution by statin use (preoperative). Figure S4. Preoperative CRP levels (mg/dL) across lipid-lowering therapy groups. Figure S5. Forest plot of β coefficients and 95% CIs from the multivariable linear model. Figure S6. Effect of spirometry phenotype on postoperative CRP (β coefficients with direction and magnitude). Figure S7. BMI versus change in CRP (scatter with regression line). Figure S8. Slope plot of pre- to postoperative CRP change with CPAP use. File S1. STROBE checklist. File S2. Pre-registered Statistical Analysis Plan (OSF DOI).

## Abbreviations

The following abbreviations are used in this manuscript:

OSA	Obstructive Sleep Apnea
CRP	C-Reactive Protein
BMI	Body Mass Index
CPAP	Continuous Positive Airway Pressure
PSM	Propensity Score Matching
CABG	Coronary Artery Bypass Grafting
AVR	Aortic Valve Replacement
MVR	Mitral Valve Replacement
EF	Ejection Fraction
HDL	High-Density Lipoprotein
LDL	Low-Density Lipoprotein
IOT	Intubation Time
IPPV	Intermittent Positive Pressure Ventilation
SIMV	Synchronized Intermittent Mandatory Ventilation
AIRVO	High-flow nasal therapy system by Fisher & Paykel
POAF	Postoperative Atrial Fibrillation
AUC	Area Under the Curve
ROC	Receiver Operating Characteristic
ΔCRP	Change in CRP (Postoperative–Preoperative)
SMD	Standardized Mean Difference
SAP	Statistical Analysis Plan
STROBE	Strengthening the Reporting of Observational Studies in Epidemiology
AASM	American Academy of Sleep Medicine
CV	Coefficient of Variation
hsCRP	High-Sensitivity C-Reactive Protein
SD	Standard Deviation
IQR	Interquartile Range
OR	Odds Ratio
VIF	Variance Inflation Factor
RCRP	CRP Extended Range Assay (Dimension Clinical Chemistry System)
