# Supplementary material for: Reevaluating C-Reactive Protein for Perioperative Risk Stratification: The Overlooked Role of Sleep Apnea in Cardiac Surgery Outcomes
Source: Biomedicines. 2025 Oct 18;13(10):2546. doi: 10.3390/biomedicines13102546 (PMC12562216; doi:10.3390/biomedicines13102546)
Supplement: Supplementary file 1 [file biomedicines-13-02546-s001.zip › Table S4. Multivariable logistic regression model assessing predictors of new-onset postoperative atrial fibrillation (AF), e.pdf]

Regression Coefficients

Predictor	Coefficient (β)	Std. Error	z-value	p-value	95% CI (Lower – Upper)
Intercept	-2.3960	2.0149	-1.1891	0.2344	-6.3451 – 1.5531
CRP (mg/dL)	-0.0022	0.0292	-0.0736	0.9413	-0.0594 – 0.0551
Sleep Apnea Severity	0.8456	0.2937	2.8788	0.0040	0.2699 – 1.4213
Age	0.0097	0.0247	0.3946	0.6931	-0.0386 – 0.0581
BMI	-0.0021	0.0444	-0.0483	0.9615	-0.0891 – 0.0848

Supplementary Table S4. Multivariable logistic regression model assessing predictors of new-onset postoperative atrial fibrillation (AF), excluding patients with preoperative AF.
The model included sleep apnea severity (ordinal score), preoperative CRP (mg/dL), age, BMI, diabetes, hypertension, and smoking status as covariates. Sleep apnea severity remained a significant independent predictor of postoperative AF (β = 0.85, p = 0.004), whereas CRP and other covariates did not reach statistical significance. The table reports regression coefficients (β), standard errors, z-values, p-values, and 95% confidence intervals. Model performance: Nagelkerke $R^2 = 0.21$; AUC = 0.74.
