# Supplementary figures and images for: Reevaluating C-Reactive Protein for Perioperative Risk Stratification: The Overlooked Role of Sleep Apnea in Cardiac Surgery Outcomes
Source: Biomedicines. 2025 Oct 18;13(10):2546. doi: 10.3390/biomedicines13102546 (PMC12562216; doi:10.3390/biomedicines13102546)

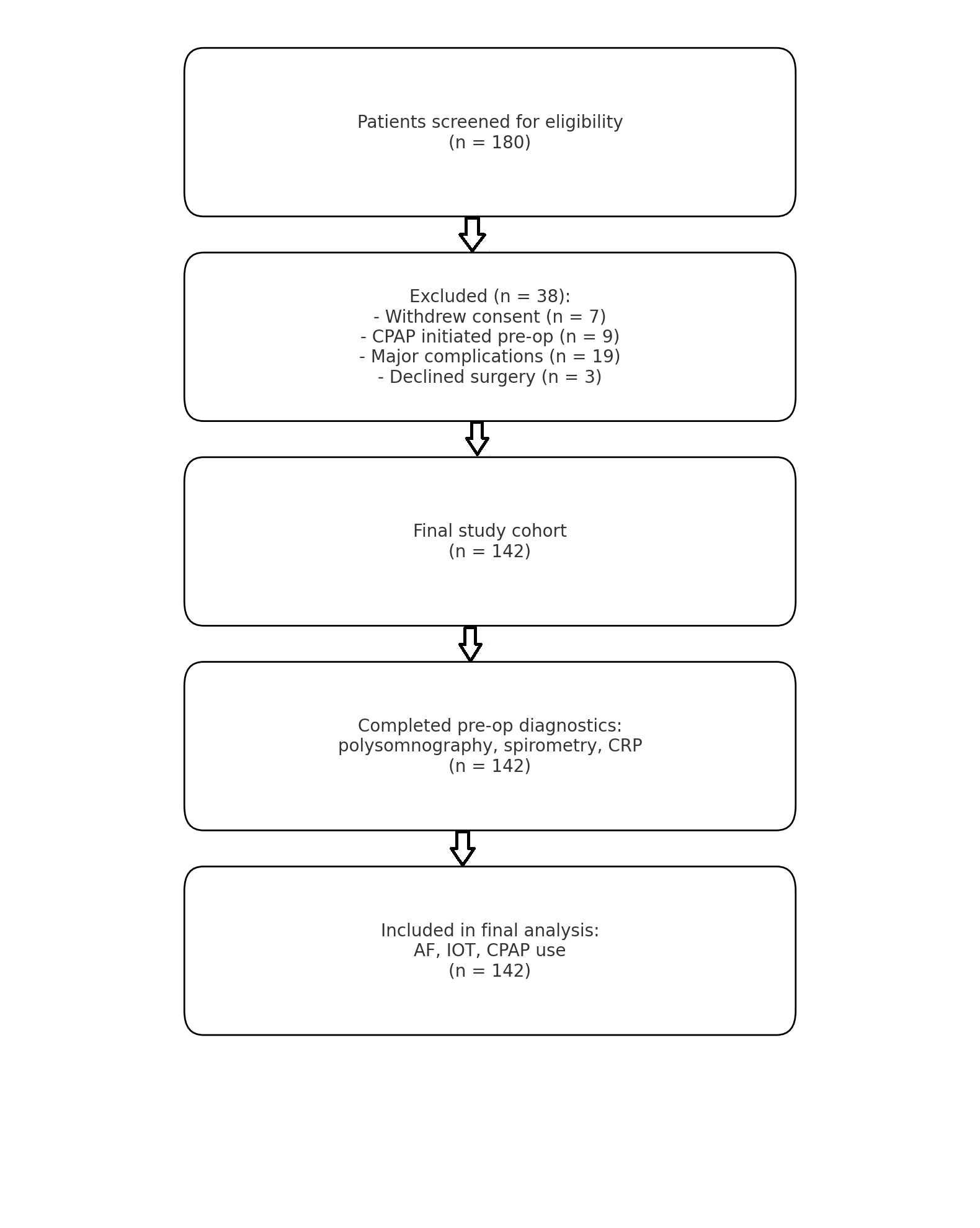

Supplement: Supplementary file 1 [file biomedicines-13-02546-s001.zip › Figure S1. Participant flow diagram illustrating screening, exclusion, and final analysis cohort (n = 142)..png]

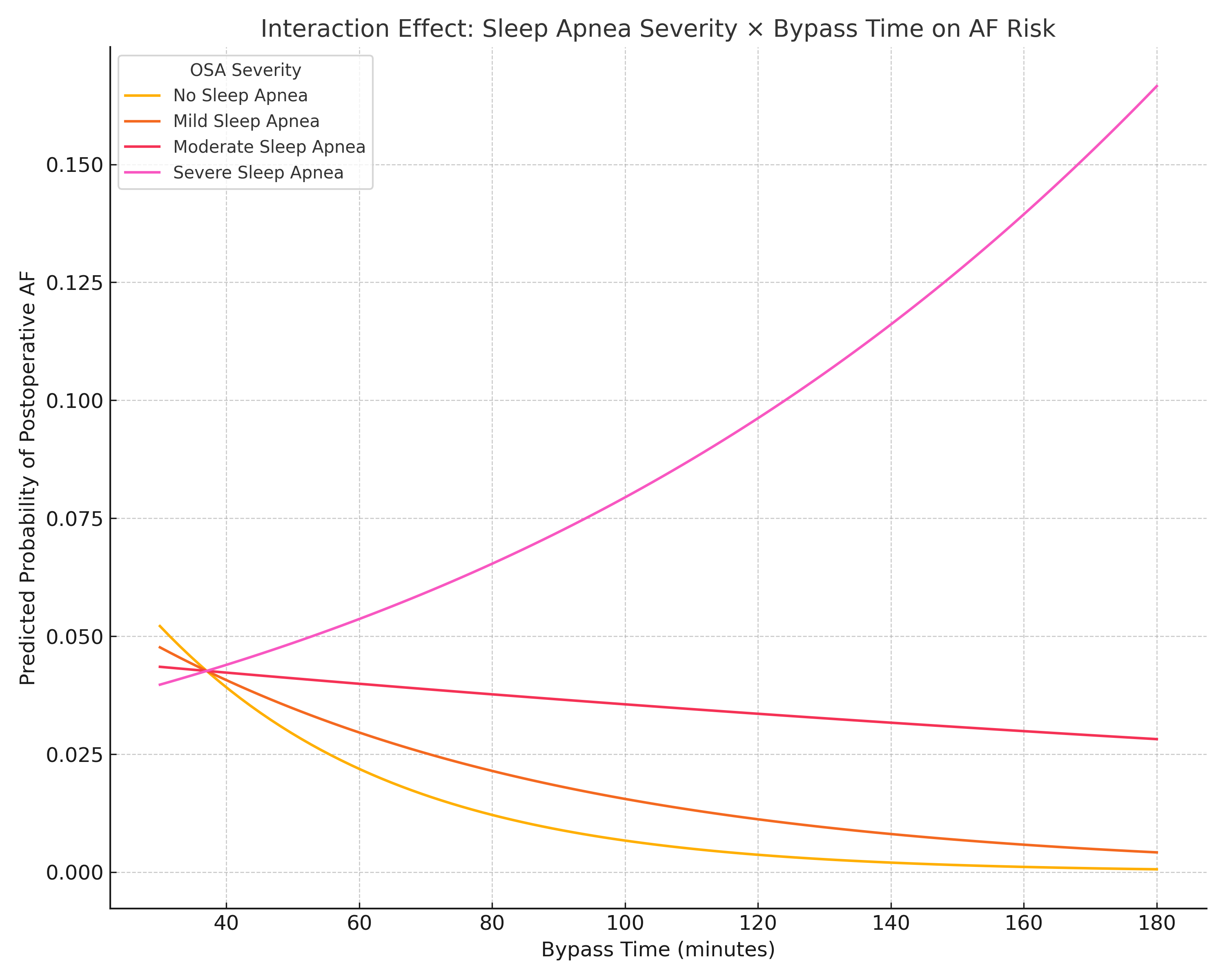

Supplement: Supplementary file 1 [file biomedicines-13-02546-s001.zip › Figure S2. Interaction effect between sleep apnea severity and bypass time on postoperative atrial fibrillation risk..png]

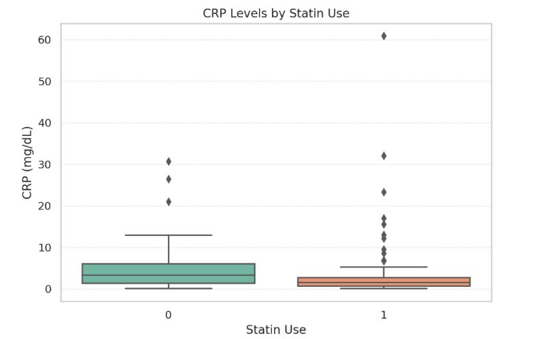

Supplement: Supplementary file 1 [file biomedicines-13-02546-s001.zip › Figure S3. Boxplot showing the distribution of C-reactive protein levels in patients with and without documented statin use..png]

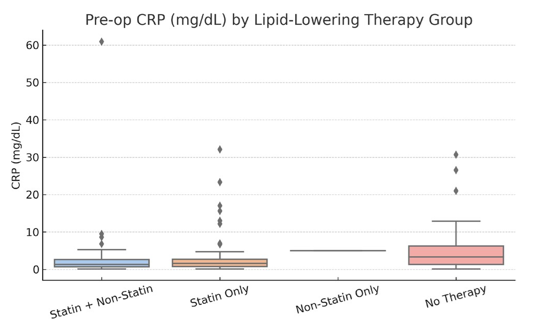

Supplement: Supplementary file 1 [file biomedicines-13-02546-s001.zip › Figure S4. Boxplot showing the distribution of preoperative CRP levels (mgdL) across four lipid-lowering therapy groups Stati.png]

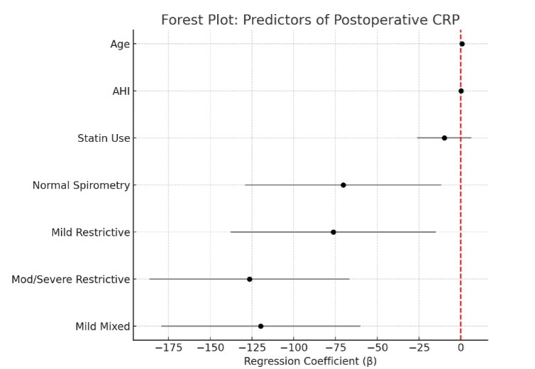

Supplement: Supplementary file 1 [file biomedicines-13-02546-s001.zip › Figure S5. Forest plot showing regression coefficients (a┬) and 95% confidence intervals from the multivariable linear model p.png]

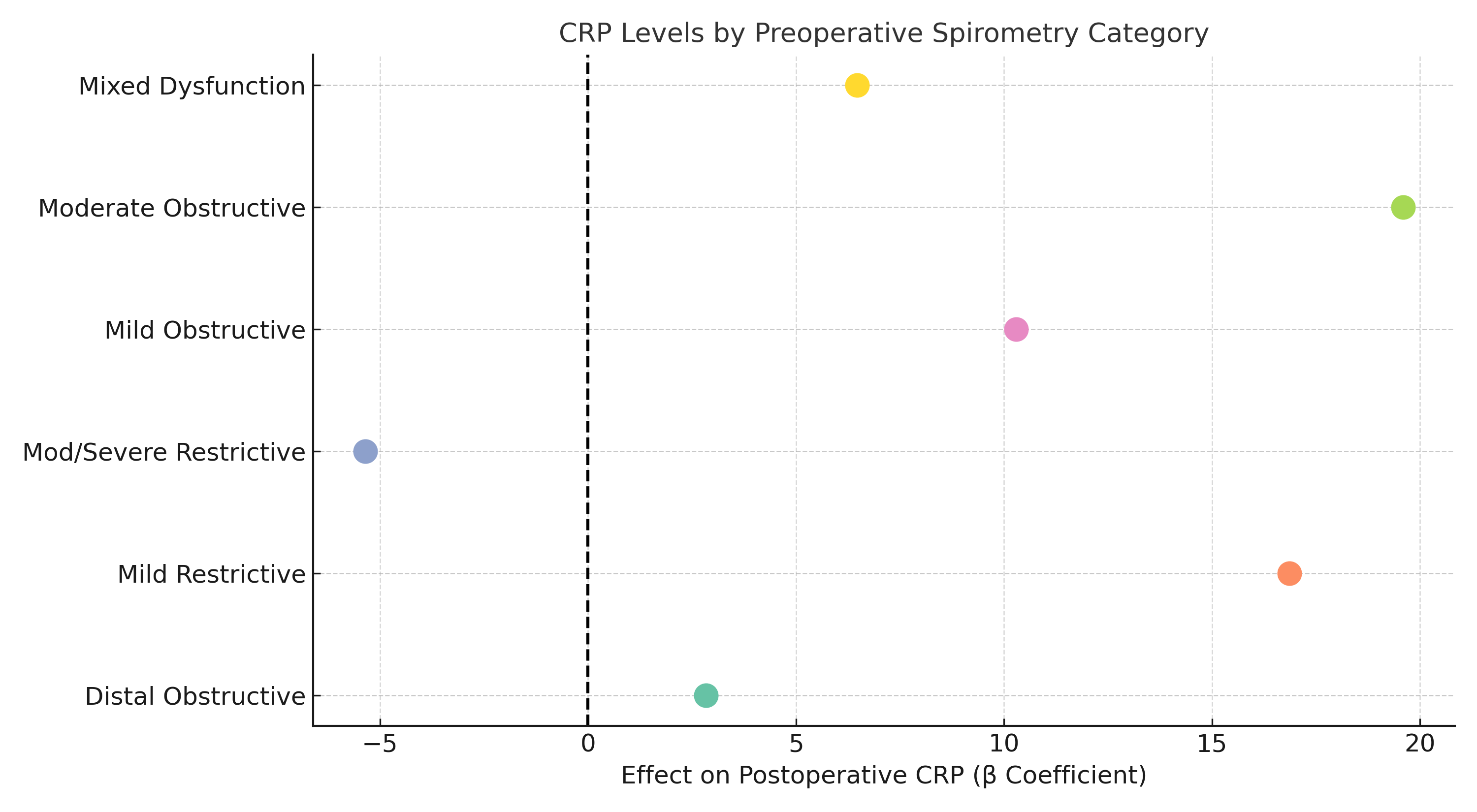

Supplement: Supplementary file 1 [file biomedicines-13-02546-s001.zip › Figure S6. Effect of preoperative spirometry phenotype on postoperative CRP (a┬ coefficients with directionality and magnitude)..png]

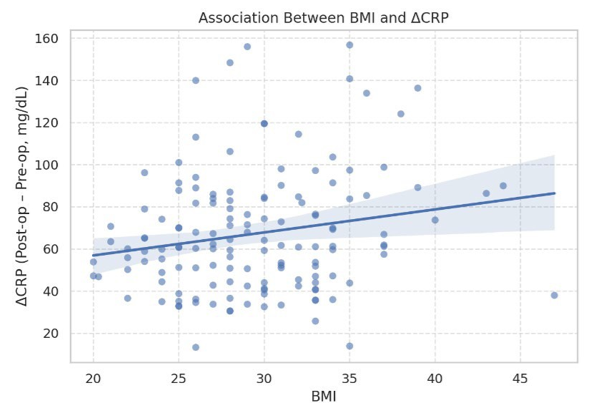

Supplement: Supplementary file 1 [file biomedicines-13-02546-s001.zip › Figure S7. Scatterplot with regression line showing the relationship between body mass index and change in C-reactive protein.png]

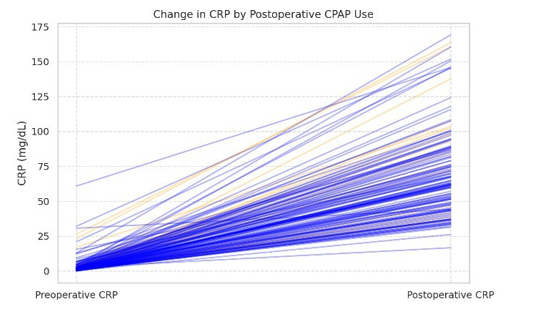

Supplement: Supplementary file 1 [file biomedicines-13-02546-s001.zip › Figure S8. Slope plot showing the change in C-reactive protein levels from preoperative to postoperative measurements in pati.png]
